# Integrating Environmental and Social Sustainability Into Performance Evaluation: A Balanced Scorecard-Based Grey-DANP Approach for the Food Industry

**DOI:** 10.3389/fnut.2018.00065

**Published:** 2018-07-20

**Authors:** Gazi M. Duman, Murat Taskaynatan, Elif Kongar, Kurt A. Rosentrater

**Affiliations:** ^1^Department of Technology Management, University of Bridgeport, Bridgeport, CT, United States; ^2^College of Doctoral Studies, Grand Canyon University, Phoenix, AZ, United States; ^3^Departments of Mechanical Engineering and Technology Management, University of Bridgeport, Bridgeport, CT, United States; ^4^Department of Agricultural & Biosystems Engineering, Iowa State University, Ames, IA, United States

**Keywords:** sustainability, food industry, performance evaluation, balanced scorecard, grey systems theory, DEMATEL, ANP

## Abstract

In addition to retaining high levels of customer satisfaction, sustainability of businesses is also heavily reliant on the efficiency of their internal and external processes. Continuous performance evaluations using key performance metrics to leverage operations are essential in maintaining a sustainable business while achieving growth objectives for revenue and profitability. Traditionally, companies have considered various financial criteria, quality characteristics, and targeted levels of service as their primary factors for performance evaluation. However, increasing environmental and social awareness and accompanying governmental legislations are now requiring companies to integrate these two aspects into their performance evaluations. With this motivation, this study proposes a Balanced Scorecard (BSC)-based approach combining Decision-Making Trial and Evaluation Laboratory (DEMATEL) and Analytic Network Process (ANP) methodologies for performance evaluation. The grey system theory has been utilized in order to capture the vagueness and the uncertainty in decision making. To demonstrate the functionality of the approach, a case study is conducted on a U.S.-based food franchise. The results of the algorithm and a discussion elaborating on the findings are provided.

## Introduction

The food industry has always been one of the essential contributors to the United States economy. The USDA (U.S. Department of Agriculture) Economic Research Services stated that in 2016, with a 12.6% share, the food and related industries ranked third after housing (33%) and transportation (15.8%) in a typical American household's expenditures (1). According to the U.S. Bureau of Economic Analysis report published in the same year, the contribution of the food services and drinking places contributed to the Gross Domestic Product (GDP) 400.7 billion dollars (2). With a 227.3 billion dollar revenue in 2016, the fast food industry obtained the highest share in this market with an expect annual growth rate of 1.8% in the following 5 years (3).

Although the fast food restaurant sector is expected to grow at a flat rate, the industry has struggled due to the shift in consumer preferences with customers moving away from unhealthy and saturated food products over the last 5 years (3). As forecasts indicate that this healthier food trend will continue in the foreseeable future, major fast food retailers have been expanding their menus to include healthier options to prevent growing numbers of obesity, diabetes and other related health issues. Increasing focus on societal well-being has also shaped the business strategies in other aspects resulting in fast food retailers making community involvement, local supplier support, environmentally benign operations more visible in their business strategies.

For instance, the collaboration between Domino's and St. Jude Children's Research Hospital has raised more than 38 million dollars since 2004 (4). Ben and Jerry's has been using only fair trade ingredients. The company also developed a sustainability program for dairy farms in its home state, Vermont (5). Starbucks, in addition to being one of the top purchasers of renewable energy from the U.S. Environmental Protection Agency, pioneered collaborative farmer programs and activities, including Coffee and Farmer Equity (C.A.F.E.). Practices, farmer support centers, farmer loans and forest carbon projects is another example of such efforts (6).

Business operations has also seen a significant impact as a result of sustainability. The triple bottom line, a.k.a. environment, people, and revenue, are now considered to be integral parts of daily operational decisions since they are vital for business success. Therefore, measuring and evaluating environmental and social sustainability indicators are as crucial as the operational and financial ones in performance assessments.

The locavore strategy aims at encouraging consumers to purchase locally grown and sold food. The green image is an indicator of the overall perception regarding environmental friendly activities. Most performance evaluation methods fall short in addressing several sustainability aspects such as supporting locavore strategies and promoting green image. Furthermore, most studies utilize conventional Analytic Hierarchy Process (AHP) methodologies where only independent and hierarchical criteria are considered and do not include the interdependencies and interactions among the decision criteria. With this motivation, this study presents a Balanced Scorecard-based holistic performance evaluation framework that integrates environmental and social sustainability criteria into performance evaluation. Proposed approach measures the influences of each criterion on others leading to more reliable and accurate assessments. This novel approach extracts the weights of main and sub-criteria without requiring additional pairwise comparisons. Uncertainty and vagueness are also additional factors that are considered in the model.

The paper has the following structure. The literature review, providing information regarding the related work, is provided in section Literature Review. In this section, the focus is on the performance evaluation practices in food industry, the Balanced Score Card (BSC), Decision-Making Trial and Evaluation Laboratory (DEMATEL), and Analytic Network Process (ANP) methodologies, and Grey Systems Theory, respectively. Section Problem Description provides the problem description. A detailed explanation of the proposed methodology is presented in section Materials and Method. The practical application of the methodology is delineated in section A Food Industry Case Study with the help of a food industry case study. Conclusions and the implications of the work for future research are given in section Conclusions and Discussion.

## Literature review

A sustainable business contributes to sustainability by delivering economic, social, and environmental benefits simultaneously (7). To ensure their longevity, these deliverables need to be continuously measured and evaluated through periodical audits (8). Thus, similar to other service industries, integrating environmental, and social sustainability measures into performance evaluation has also become a necessity in the food industry. Motivated by this need, Gerbens-Leenes et al. (9) presented the findings regarding the use of environmental indicators for food production and proposed a method for measuring the environmental sustainability in food production systems. Salvá et al. (10) developed an audit tool for environmental measurement in the UK food sector. Maloni et al. (11) presented a detailed framework of unique Corporate Social Responsibility (CSR) applications in the food supply chain including animal welfare, biotechnology, environment, fair trade, health, and safety, and labor and human rights. Furthermore, in her study, Hartmann et al. (12) connected the rich body of literature on CSR to the food sector.

The balanced scorecard is a widely used strategic planning tool for performance measurement where financial and non-financial measures are integrated with corporate visions. In the literature, a variety of studies apply BSC to various fields including finance, human resources, supply chain management, sales, and marketing, and so on in order to pursue an effective and efficient visionary improvement of an organization. Even though the BSC model is proposed by Kaplan and Norton in 1992 (13) is more applicable for the operations of the profit-oriented organizations, the BSC model is also applicable for the socially and environmentally concerned processes, where particular characteristics of these processes that give emphasis on how well the organization fulfills its mission is considered (14–16).

Several studies on BSC also involve sustainability approach in order to implement the performance evaluation center around multi-criteria decision-making approaches. Kongar (17) proposed a Green BSC approach combining with Linear Physical Programming (LPP) to measure the performance of supply chain management while defining the appropriate measurement criteria. Tsai et al. (18) utilized the sustainability balanced scorecard as a multi-criteria framework for socially responsible investment evaluation. Hsu et al. (19) utilized Fuzzy Delphi Method and ANP to construct a sustainability balanced scorecard framework to measure the sustainable performance for the semiconductor industry in Taiwan. Bhattacharya et al. (20) used a using fuzzy ANP-based balanced scorecard to determine a green supply chain performance measurement framework. Rabbani et al. (21) integrated sustainability balanced scorecard, ANP, and COPRAS (Complex Proportional Assessment) techniques to evaluate the performance of oil producing companies in Iran.

Decision-Making Trial and Evaluation Laboratory (22, 23) and Analytic Network Process (ANP) (24) are two well-studied methodologies in the Multi-Criteria Decision Making (MCDM) field (25). Various combinations of these two approaches have also been developed to determine the influences and interdependence among the evaluation criteria (26). The DEMATEL-based ANP (DANP) is the general form of cluster-weighted ANP. Traditional ANP requires the unweighted super-matrix to be built based on pairwise comparisons. The criteria weights are then obtained by limiting this super-matrix. In order to avoid the need for additional pairwise comparison data, DANP forms a comprehensive unweighted super-matrix by building the direct influence matrix where pairwise comparisons are included within clusters. After the unweighted super-matrix is built, the total relation matrices between clusters are utilized to construct the weighted super-matrix. Details of the approach in addition to its various applications can be found in Chen et al. (27), Chiu et al. (28), Hsu et al. (29), Hung et al. (30), Lee et al. (31), Liou (32), Wu, (33), Wu and Lee (34).

Uncertainty and incomplete information are two issues commonly encountered in multi-criteria decision making environment. Decision makers tend to use linguistic preference relations to express their preferences where there is lack of information (i.e., lack of numerical values for comparison). However, these linguistic preferences usually contain uncertainty and vagueness in the decision making process. Grey system theory (35) can be utilized to address these uncertainty issues. It provides an approach for analysis and modeling of systems with limited and incomplete information, and which may exhibit random uncertainty (36). Grey system theory research areas contain systems analysis, data processing, modeling, prediction, as well as decision making (37, 38). In their literature survey, Tozanli et al. (39) stated that the total number of studies incorporating grey system theory has increased significantly over the past 5 years including the publications in sustainability and MCDM fields. Golmohammadi et al. (40), developed a two-phased grey decision making approach to the supplier selection. Fu et al. (36) applied a grey DEMATEL approach to evaluate the green supplier development programs at a telecommunication systems provider. Furthermore, Dou et al. (37) used a grey-ANP method to identify green supplier development programs. Chithambaranathan et al. (41) applied a grey based hybrid MCDM framework for evaluating the environmental performance of service supply chains. Çelikbilek and Tüysüz (42) proposed an integrated grey MCDM approach for the evaluation of renewable energy sources.

A review of the existing literature reveals that no earlier study combining Grey System Theory, BSC, and DANP methodologies has been proposed to integrate environmental and social sustainability criteria into performance evaluation in the food industry. To the best of our knowledge, this study is the only research determines the weights of influenced criteria in franchised food retail stores via utilizing the methods mentioned above.

## Problem description

Increasing awareness of environmental and social sustainability has resulted in many companies making significant investments to integrate these measures into their performance evaluations. Determining the performance criteria and the criteria weights based on the particular industry in focus have been the subject of several studies in the literature. Compared to conventional manufacturing industries, establishing appropriate measures for service industries is a more challenging task. Particularly, as mentioned in section Introduction, fast food restaurant businesses are striving hard to implement various sustainable practices in that are suitable for the specific operations. Therefore, there is a need for an approach capable of considering the specific needs of businesses when defining the sustainability measures for that particular business.

Traditional AHP is highly capable of obtaining criteria weights via pairwise comparison in a hierarchical structure. However, the contemporary research in multi-criteria decision-making suggests that the evaluation criteria are influenced by one another and hence need to be represented as a network instead of hierarchically. With this motivation, this study propose a Balanced Scorecard-based DANP approach to determine the weights of the criteria. A case study in the food industry is also presented to illustrate the applicability of the proposed model.

## Materials and method

**Figure 1:**
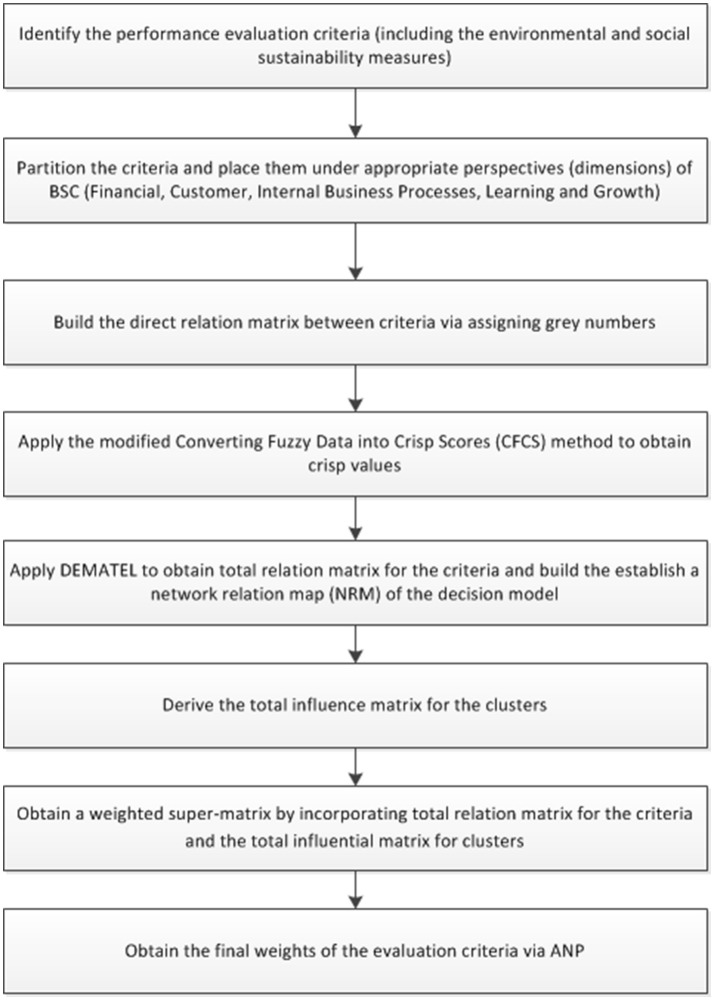
Schematic representation of the proposed methodology.

Kaplan and Norton (43) stated that the interrelation between the four perspectives of a typical Balanced Scorecard could be represented by a strategy map. Strategy map is a blueprint any organization can follow to align processes, people, and information technology for higher performance. Therefore, this study utilizes a Balanced Scorecard based Grey-DANP approach to determine the appropriate weights of the evaluation criteria. Figure 1 provides the steps of the methodology. The details of the methodology are provided in the following.

### Grey system theory

Grey system theory was first introduced by Deng et al. (35) to deal with insufficient and incomplete information. In grey systems theory, a system is called a *white system* if the system information is fully known; and a *black system* if the information is not known at all. A system with partially known information is called a *grey system*. A grey number is a number with uncertain and/or incomplete information and can be mathematically expressed as $⊗X=\left[\underline{X}\;,\bar{X}\right]=\{X|\;\underline{X}\leq X\leq \bar{X},\;\underline{X}\;and\;\bar{X}\;\epsilon \;R\}$. Thus, ⊗*X* contains two real numbers X (the lower limit of ⊗*X*) and $\bar{X}$ (the upper limit of ⊗*X*) is defined as below:

- If $\underset{-}{X}\to \;-\infty and\;\bar{X}\to \;\infty \;,$ then ⊗*X* is a black number with no meaningful information,
- Else if $\underset{-}{X}=\bar{X}$, then ⊗*X* is a white number with complete information,
- Otherwise $⊗X=\left[\underset{-}{X}\;,\bar{X}\right]$, ⊗*X* is a grey number with insufficient and/or uncertain information.

Let there be two sets of grey numbers denoted by $⊗X_{1}=\left[\underline{X_{1}}\;,\bar{X_{1}}\right]\text{ and }⊗X_{2}=\left[\underline{X_{2}}\;,\bar{X_{2}}\right]$. The basic mathematical operations for these two sets of grey numbers are listed below.

(1) $$\begin{array}{l} ⊗X_{1}+⊗X_{2}=\left[\underline{X_{1}}+\underline{X_{2}},\bar{X_{1}}+\bar{X_{2}}\right] \end{array}$$

(2) $$\begin{array}{l} ⊗X_{1}-⊗X_{2}=\left[\underline{X_{1}}-\bar{X_{2}},\bar{X_{1}}-\underline{X_{2}}\right] \end{array}$$

(3) $$\begin{array}{l} ⊗X_{1}*⊗X_{2}=[min(\underline{X_{1}}\underline{X_{2}},\bar{X_{1}}\bar{X_{2}},\underline{X_{1}}\bar{X_{2}},\bar{X_{1}}\underline{X_{2}}),\\ \;\;\;\;\;\;\;\;\;\;\;\;\;\;\;\;\;\;\;\;\;\;max(\underline{X_{1}}\underline{X_{2}},\bar{X_{1}}\bar{X_{2}},\underline{X_{1}}\bar{X_{2}},\bar{X_{1}}\underline{X_{2}})] \end{array}$$

(4) $$\begin{array}{l} ⊗X_{1}:⊗X_{2}=\left[\underline{X_{1}},\bar{X_{1}}\right]*[\frac{1}{\bar{X_{2}}},\frac{1}{\underline{X_{2}}}] \end{array}$$

(5) $$\begin{array}{l} k^{*}⊗X_{1}=\left[k\underline{X_{1}},k\bar{X_{1}}\right],k\epsilon R \end{array}$$

(6) $$\begin{array}{l} {⊗X_{1}}^{-1}=[\frac{1}{\bar{X_{1}}},\frac{1}{\underline{X_{1}}}] \end{array}$$

In order to deal with the problems in a grey environment, an effective whitenization (grey aggregation) method is required. Opricovic et al. (44) developed Converting Fuzzy Data into Crisp Scores (CFCS) method to cope with the uncertainty and vagueness in multi-criteria decision problems. CFCS is designed to distinguish between two fuzzy numbers with the same crisp value obtained by the Centroid (center of gravity) method independent of the shape of the fuzzy numbers (34). The steps of the modified CFCS method utilized in this research are provided below.

Let $⊗\;x_{ij}^{p}=\left[\underline{x_{ij}^{p}}\;,\bar{x_{ij}^{p}}\right]$ indicate the grey assessment of evaluator, *p* (decision maker), that will evaluate the influence of criterion *i* on criterion *j*. Then the following

Step 1: Normalization

(7) $$\begin{array}{l} \tilde{\bar{x_{ij}^{p}}}=\left[\bar{x_{ij}^{p}}-min_{j}\bar{x_{ij}^{p}}\right]/\Delta_{\min}^{\max} \end{array}$$

(8) $$\begin{array}{l} \tilde{\underline{x_{ij}^{p}}}=\left[\underline{x_{ij}^{p}}-min_{j}\underline{x_{ij}^{p}}\right]/\Delta_{\min}^{\max} \end{array}$$

where

(9) $$\begin{array}{l} \Delta_{\min}^{\max}=max_{j}\bar{x_{ij}^{p}}-min_{j} \end{array}$$

Step 2: Determination of a total normalized crisp value

(10) $$\begin{array}{l} Y_{ij}^{p}=\frac{\left(\tilde{\underline{x_{ij}^{p}}}(1-\tilde{\underline{x_{ij}^{p}}})+(\tilde{\bar{x_{ij}^{p}}}*\tilde{\bar{x_{ij}^{p}}})\right)}{\left(1-\tilde{\underline{x_{ij}^{p}}}+\tilde{\underline{x_{ij}^{p}}}\right)} \end{array}$$

Step 3: Calculate crisp values

(11) $$\begin{array}{l} z_{ij}^{p}=min_{j}x_{ij}^{p}+Y_{ij}^{p}\Delta_{\min}^{\max} \end{array}$$

### The DEMATEL based ANP (DANP) method

The DANP is a novel approach that combines the original DEMATEL and ANP methods to utilize total relation matrix for the criteria and the clusters, viz., the BSC dimensions in this study, and to build a network relation map (NRM) of the decision model. Based on the network relation map, the influential relationships are then obtained (26). The basic steps of the DANP approach are provided as follows:

- Generate the direct relation matrix

The initial step in this process is to obtain the decision maker assessments regarding the direct influence among the criteria. These assessments are represented as grey numbers and can be represented by one of the following five levels; “no influence,” “low influence,” “medium influence,” “high influence,” and “very high influence,” Here, the initial direct-relation matrix ***A*** is an *n* × *n* matrix where *a*_*ij*_ indicates the degree that the criterion *i* affects the criterion *j* and *A* = [_*a*_*ij*_]*nxn*_.

- Normalize the direct relation matrix

The normalized direct-relation matrix *X* = [_*x*_*ij*_]*nxn*_ can be obtained through

(12) $$\begin{array}{l} X=A/s \end{array}$$

(13) $$\begin{array}{l} where\;s=max\left[max\sum_{i\;=\;1}^{n}a_{ij},max\sum_{j\;=\;1}^{n}a_{ij}\right]. \end{array}$$

Here, the normalized initial direct-relation matrix is obtained via Equation (12), and the ***s*** value representing the maximum values of the sums of all the rows and the sums of all the columns is calculated via Equation (13).

- Obtain the total relation matrix

The total relation matrix ***T*** = [_*t*_*ij*_]*nxn*_ can be obtained by utilizing Equation (14) where ***I*** is the identity matrix:

(14) $$\begin{array}{l} T=X+X^{2}+X^{3}+\ldots +X^{k}=\sum_{k\;=\;1}^{\infty }X^{k}=X{\left(I-X\right)}^{-1}. \end{array}$$

Furthermore, the method utilizes the sums of each row and column of the matrix *T* to build the NRM.

(15) $$\begin{array}{l} d_{i}={\left(r_{i}\right)}_{nx1}={\left[\sum_{j\;=\;1}^{n}t_{ij}\right]}_{nx1}. \end{array}$$

(16) $$\begin{array}{l} r_{j}={\left(c_{j}\right)}_{nx1}={\left[\sum_{i\;=\;1}^{n}t_{ij}\right]}_{1xn}. \end{array}$$

Here, Equation (15) represents the row sum of the *i*th row of matrix *T* and shows the sum of direct and indirect effects of criterion *i* on the other criteria. Similarly, Equation (16) represents the sum of the *j*th column of matrix *T* and shows the sum of direct and indirect effects that criterion *j* has received from the other criteria. Furthermore, (*d*+*r*) indicates the importance of the criterion. Here, if (*d–r*) results in positive value it is implied that the criterion has an effect on others. Similarly, when (*d–r*) obtains a negative value then the criterion is affected by the others.

- Formation of an unweighted super-matrix

The weighted super-matrix is obtained by dividing each element in a column by the number of clusters with each cluster having equal weights. However, the equal weight assumption for each cluster is not always feasible due to the different degrees of influence among the criteria (45). In order to relax this unrealistic assumption, two different total influence matrices are then utilized. The first one, $T_{c}={\left[t_{c}^{ij}\right]}_{nxn}$ pertains to *m* criteria, while the second one, $T_{D}={\left[t_{D}^{ij}\right]}_{nxn}$ is devoted to *n* dimensions, i.e., clusters, as shown in Equations (17) and (18). 
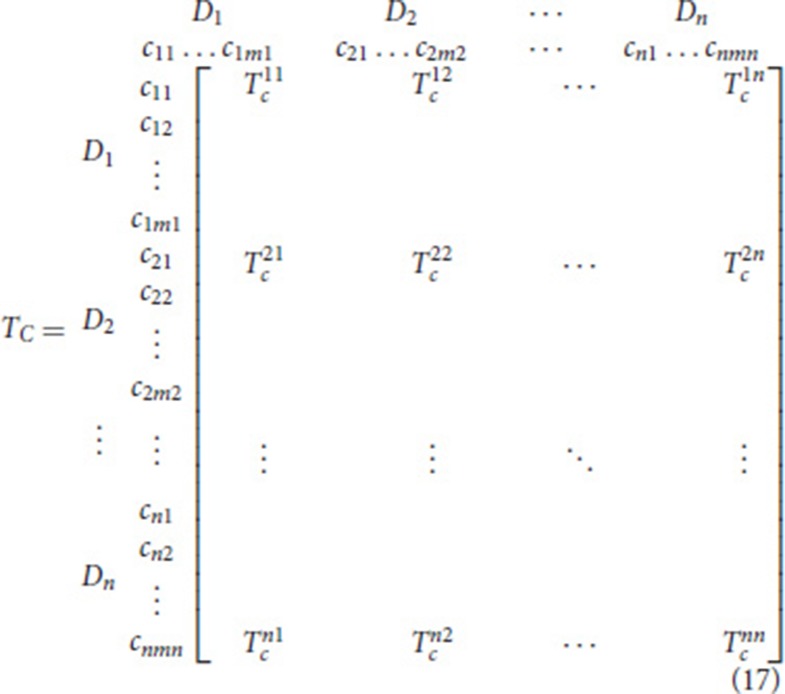


(18) $$\begin{array}{l} T_{D}=\left[\begin{array}{ccccc} t_{D}^{11} & \cdots & t_{D}^{1j} & \cdots & t_{D}^{1n}\\ \vdots & \; & \vdots & \; & \vdots \\ t_{D}^{i1} & \cdots & t_{D}^{ij} & \cdots & t_{D}^{in}\\ \vdots & \; & \vdots & \; & \vdots \\ t_{D}^{n1} & \cdots & t_{D}^{nj} & \cdots & t_{D}^{nn} \end{array}\right] \end{array}$$

- Normalize the total relation and total influence matrices

The normalized total relation matrix of criteria $T_{C}^{nor}$ is computed by dividing the sum of each row in each sub-matrix. For instance, the normalized sub-matrix $T_{C}^{nor12}$ is calculated as in Equation (19). 
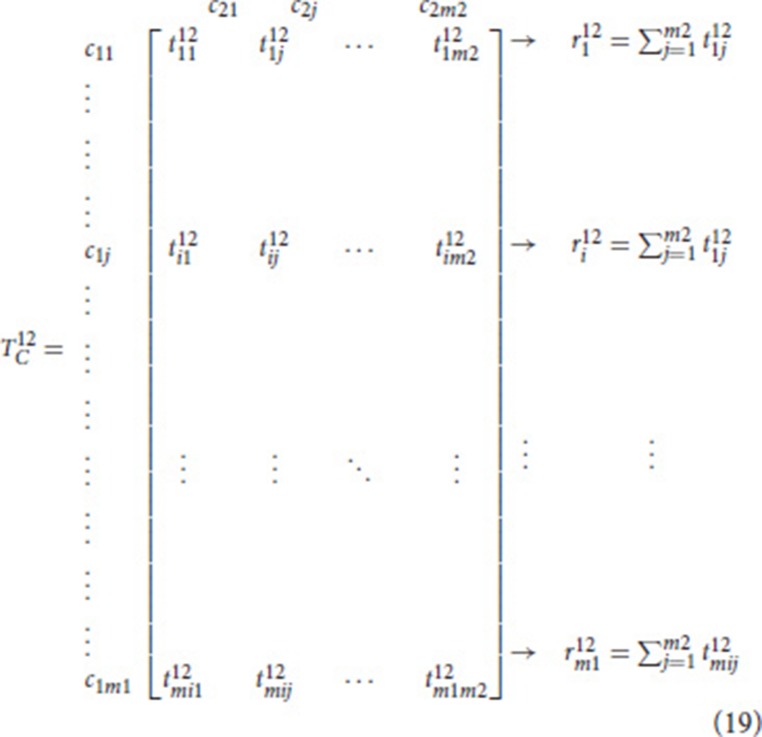


where $r_{i}^{12}$ represents the sum of each row in the sub matrix $T_{C}^{12}$. Then $T_{C}^{nor12}$ is obtained as shown in Equation (20).

(20) $$\begin{array}{l} T_{C}^{nor12}=\left[\begin{array}{cccc} t_{11}^{12}/r_{1}^{12} & t_{1j}^{12}/r_{1}^{12} & \cdots & t_{1m2}^{12}/r_{1}^{12}\\ t_{i1}^{12}/r_{i}^{12} & t_{ij}^{12}/r_{i}^{12} & \cdots & t_{im2}^{12}/r_{i}^{12}\\ \vdots & \vdots & \ddots & \vdots \\ t_{mi1}^{12}/r_{m1}^{12} & t_{mij}^{12}/r_{m1}^{12} & \cdots & t_{m1m2}^{12}/r_{m1}^{12} \end{array}\right] \end{array}$$

Similar to $T_{C}^{nor},$ the normalized total influential matrix for clusters $T_{D}^{nor}$ is formed as shown in equation (21).

(21) $$\begin{array}{l} T_{D}^{nor}=\left[\begin{array}{ccccc} t_{D}^{11}/t_{D}^{1} & \cdots & t_{D}^{1j}/t_{D}^{1} & \cdots & t_{D}^{1n}/t_{D}^{1}\\ \vdots & \vdots & \vdots \\ t_{D}^{i1}/t_{D}^{i} & \cdots & t_{D}^{ij}/t_{D}^{i} & \cdots & t_{D}^{in}/t_{D}^{i}\\ \vdots & \vdots & \vdots \\ t_{D}^{n1}/t_{D}^{i} & \cdots & t_{D}^{nj}//t_{D}^{i} & \cdots & t_{D}^{nn}/t_{D}^{i} \end{array}\right]=\left[\begin{array}{ccccc} t_{D}^{nor11} & \cdots & t_{D}^{nor1j} & \cdots & t_{D}^{nor1n}\\ \vdots & \vdots & \vdots \\ t_{D}^{nori1} & \cdots & t_{D}^{norij} & \cdots & t_{D}^{norin}\\ \vdots & \vdots & \vdots \\ t_{D}^{norn1} & \cdots & t_{D}^{nornj} & \cdots & t_{D}^{nornn} \end{array}\right] \end{array}$$

where the sum of each cluster is defined as $t_{D}^{i}=\sum_{j\;=\;1}^{n}t_{D}^{ij}.$

- Build a weighted super-matrix

The unweighted super-matrix *U*_*C*_ is the matrix transposed from the normalized total relation matrix for the criteria $T_{C}^{nor}$ as shown in Equation (22). 
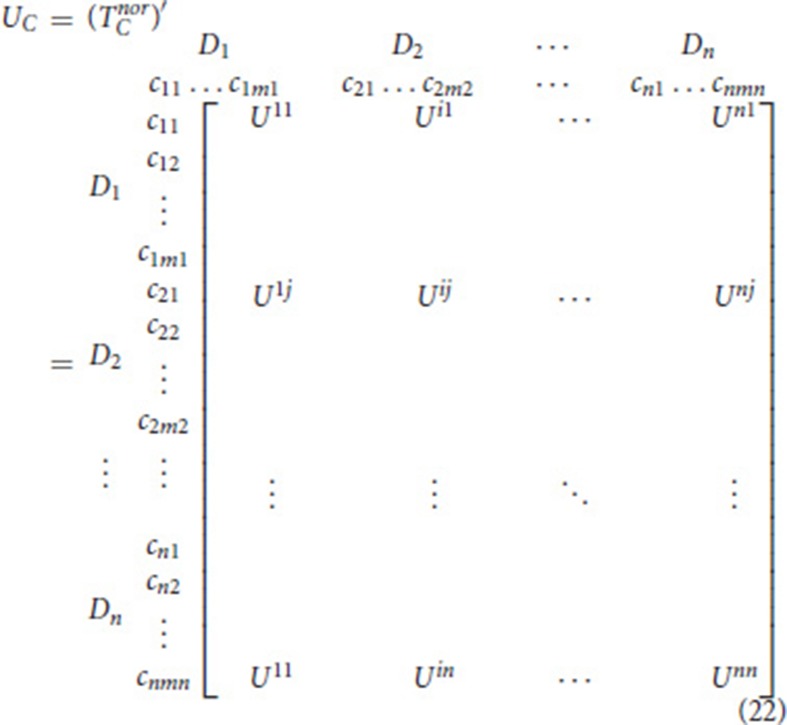


The weighted super-matrix *W* is obtained by incorporating the unweighted super-matrix *U*_*C*_ and the normalized total influential matrix for clusters $T_{D}^{nor}$ is shown in Equation (23):

(23) $$\begin{array}{l} W=\left[\begin{array}{ccccc} t_{D}^{nor11}xU^{11} & \cdots & t_{D}^{nori1}xU^{i1} & \cdots & t_{D}^{nor1n}xU^{n1}\\ \vdots & \vdots & \vdots \\ t_{D}^{nor1j}xU^{1j} & \cdots & t_{D}^{norij}xU^{ij} & \cdots & t_{D}^{norinj}xU^{nj}\\ \vdots & \vdots & \vdots \\ t_{D}^{nor1n}xU^{1n} & \cdots & t_{D}^{nornj}xU^{in} & \cdots & t_{D}^{nornn}xU^{nn} \end{array}\right] \end{array}$$

- Limit the weighted super-matrix to obtain criteria weights

In order to obtain the final influential criteria weights, the weighted super-matrix *W* is raised to a sufficiently large power *z* until it converges and becomes a long-term stable super-matrix:

(24) $$\begin{array}{l} \underset{s\to \infty }{\lim}{\left(W\right)}^{z} \end{array}$$

## A food industry case study

The case study is conducted in a U.S. based fast food restaurant company that owns several franchise retail stores in

the northeast region. The company management is in the process of introducing sustainability into their performance evaluation system and is investigating how these perspectives interact with each other. With this motivation, this study applies the proposed methodology using the data collected from the subject matter experts in the company.

### Criteria definition

Based on the balanced scorecard approach, the literature review, expert opinions, and interviews with the upper level management of the company, the performance evaluation criteria are determined. The criteria and their respective definitions are provided in Table 1.

**Table 1:** Criteria and their definitions.

**Dimensions**	**Criteria**	**Definition**	
Financial (*D*_1_)	*C*_11_	Weekly sales	Total weekly sales in each store
	*C*_12_	Weekly expenses	Total weekly expenses in each store
	*C*_13_	Total number of carry-out orders	Total number of weekly carry-out orders in each store
	*C*_14_.	Total number of delivery orders	Total number of weekly delivery orders in each store
arning & Growth (*D*_2_)	*C*_21_	Food safety initiatives	A measure related to increasing food safety in each store
	*C*_22_	Operational safety initiatives	A measure related to increasing safety both in-store and in delivery operations
	*C*_23_	Quality and development initiatives	A measure related to building skills and capabilities for higher product and service quality in each store
	*C*_24_	Sustainable development initiatives	A measure related to building skills and capabilities in sustainability applications integrated into the routine workflow in each store
Customer (*D*_3_)	*C*_31_	Number of customer complaints	Total number of weekly customer complaints in each store
	*C*_32_	Green image of the store	A measure related to the overall green image from a customer view in each store
	*C*_33_	Social responsibility image of the store	A measure related to the overall social responsibility image from a customer view in each store
Internal Business Processes (*D*_4_)	*C*_41_	On-time delivery ratio	The ratio of the amount of orders delivered no later than the estimated time
	*C*_42_	Out to door time ratio	The ratio of the amount of orders completed in the store no later than the estimated time
	*C*_43_	Resource utilization ratio	The ratio of the utilization of in-store personnel, delivery personnel, materials, and other resources in the store
	*C*_44_	Forecast accuracy ratio of food inventory	The ratio of forecasting accuracy of the raw food amount ordered weekly
	*C*_45_	Utilization of local food suppliers	The utilization ratio of local food suppliers in the neighborhood
	*C*_46_	Rate of proper recycling and waste disposal	The ratio of ensuring safety and protecting the environment through proper recycling and disposal of wastes

### Application of the proposed model

As mentioned in the proposed methodology section, Balanced Scorecard-Based Grey-DANP approach is applied to determine the global weights of the dimensions in BSC and the criteria. The decision makers used linguistic terms to assess the influences between the criteria. The assessment scale used in Grey-DANP is provided in Table 2.

**Table 2:** The grey linguistic scale for the assessments.

**Linguistic terms**	**Grey Numbers**
No influence (N)	[0, 0]
Low influence (L)	[0, 0.25]
Medium influence (M)	[0.25, 0.50]
High influence (H)	[0.50, 0.75]
Very high influence (VH)	[0.75, 1.00]

The direct relation matrix is formed according to franchisee and supervisors point of view and demonstrated in Table 3.

**Table 3:** The linguistic scale direct-relation matrix for the criteria.

**Criteria**	***C*_11_**	***C*_12_**	***C*_13_**	***C*_14_**	***C*_21_**	***C*_22_**	***C*_23_**	***C*_24_**	***C*_31_**	***C*_32_**	***C*_33_**	***C*_41_**	***C*_42_**	***C*_43_**	***C*_44_**	***C*_45_**	***C*_46_**
*C*_11_	N	H	VH	VH	N	N	N	N	N	N	N	VH	VH	VH	H	M	N
*C*_12_	VH	N	H	H	L	L	L	L	N	M	H	H	H	N	N	L	N
*C*_13_	VH	M	N	N	L	L	L	L	N	L	L	VH	VH	H	L	H	N
*C*_14_	VH	M	N	N	L	L	L	L	N	L	L	VH	VH	H	L	H	N
*C*_21_	N	M	N	N	N	M	H	H	H	M	M	N	N	N	M	M	M
*C*_22_	N	M	N	N	M	N	H	H	M	M	M	N	N	N	L	M	L
*C*_23_	VH	M	N	N	H	H	N	H	H	M	M	N	N	N	L	L	L
*C*_24_	N	L	N	N	H	H	H	N	L	M	M	N	N	L	L	M	M
*C*_31_	N	N	H	H	H	H	H	H	N	L	L	VH	VH	H	M	N	L
*C*_32_	N	L	L	L	M	M	M	H	N	N	H	N	N	N	L	L	VH
*C*_33_	N	L	L	L	H	M	H	H	L	VH	N	N	N	N	L	VH	VH
*C*_41_	VH	M	VH	VH	N	N	N	N	VH	N	N	N	VH	VH	L	M	N
*C*_42_	VH	M	VH	VH	N	N	N	N	VH	N	N	VH	N	VH	L	M	N
*C*_43_	VH	M	N	H	N	N	N	N	VH	M	N	VH	VH	N	L	L	L
*C*_44_	L	H	N	N	H	M	L	L	L	L	L	N	N	M	N	N	L
*C*_45_	L	M	L	L	H	L	L	H	L	L	VH	N	N	M	H	N	N
*C*_46_	N	L	N	N	L	L	M	VH	VH	VH	VH	N	N	N	N	N	N

The whitened assessments in the direct relation matrix are obtained via utilizing modified-CFCS method from Equations (7–11). Following this, the total relation matrix is obtained by utilizing Equations (12–14). The results are provided in the Appendix A in Tables A1,A2. Furthermore, the total influences given and received on the criteria along with the dimensions can be calculated using Equations (15, 16) as shown in Table 4.

**Table 4:** The sum of influences provided and received on the criteria and dimensions.

**Dimensions/Criteria**	***d*_*i*_**	***r*_*j*_**	***d*_*i*_ + *r*_*j*_**	***d*_*i*_ − *r*_*j*_**	
*D*_1_	Financial	0.689	0.692	1.381	−0.003
*C*_11_	Weekly sales	1.305	1.383	2.687	−0.078
*C*_12_	Weekly expenses	0.985	0.806	1.791	0.179
*C*_13_	Total number of carry-out orders	0.955	0.951	1.906	0.005
*C*_14_	Total number of delivery orders	0.955	1.061	2.017	−0.106
*D*_2_	Learning & Growth	0.466	0.528	0.994	−0.062
*C*_21_	Food safety initiatives	0.630	0.615	1.246	0.015
*C*_22_	Operational safety initiatives	0.557	0.546	1.102	0.011
*C*_23_	Quality and development initiatives	0.674	0.634	1.308	0.040
*C*_24_	Sustainable development initiatives	0.611	0.678	1.289	−0.067
*D*_3_	Customer	0.591	0.534	1.124	0.057
*C*_31_	Number of customer complaints	0.423	0.398	0.821	0.024
*C*_32_	Green image of the store	0.330	0.417	0.746	−0.087
*C*_33_	Social responsibility image of the store	0.469	0.406	0.875	0.063
*D*_4_	Internal Business Processes	0.629	0.621	1.251	0.008
*C*_41_	On-time delivery ratio	1.598	1.450	3.048	0.148
*C*_42_	Out to door time ratio	1.598	1.450	3.048	0.148
*C*_43_	Resource utilization ratio	1.355	1.376	2.731	−0.022
*C*_44_	Forecast accuracy ratio of food inventory	0.347	0.545	0.892	−0.197
*C*_45_	Utilization of local food suppliers	0.544	0.771	1.315	−0.227
*C*_46_	Rate of proper recycling and waste disposal	0.452	0.301	0.753	0.151

**Figure 2:**
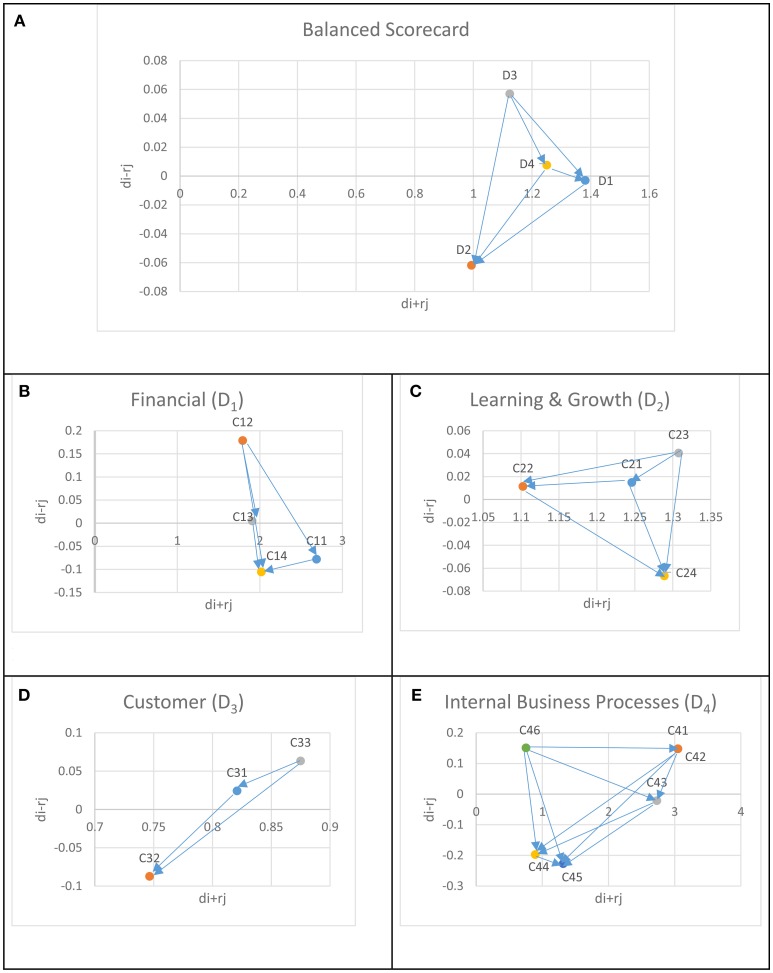
The network relation map. **(A)** The network relation map of the BSC dimensions. **(B)** The network relation map of the criteria under D1. **(C)** The network relation map of the criteria under D2. **(D)** The network relation map of the criteria under D3. **(E)** The network relation map of the criteria under D4.

Thus, the influence diagram, a.k.a. the network relation map (NRM) from the DEMATEL method can be obtained as illustrated in Figure 2.

According to the results of the total relation matrix provided in Table A2, the influential weight of each criterion is obtained via the ANP algorithm. The normalized super-matrix $T_{C}^{nor}$ is built by employing Equations (17–20). An unweighted super-matrix *U*_*C*_ can be derived by transposing the normalized matrix as shown in Equation (22). The weights of the BSC dimensions (clusters) can be derived from the improved DANP method without employing further surveys as shown in Equation (21) instead of using additional pairwise comparisons amongst dimensions (45). Therefore, the weighted super-matrix *W* can be obtained through Equation (23). The final influential weights of each criterion can then be obtained by limiting the power of the weighted super-matrix until it converges into a steady state as shown in Equation (24). Resulting matrices are provided in Appendix A from Tables A3–A5, respectively. The global weights of the criteria (Table A5) can be obtained from the DANP method. Using these results, the local weight of each criterion and dimension can also be derived accordingly. The global and local weights along with the rankings of the criteria and BSC dimensions are provided in Table 5.

**Table 5:** The influential weights of the BSC dimensions and the criteria.

**Dimension**	**Local weight**	**Ranking**	**Criteria**	**Local weight**	**Global weight**	**Ranking**
D_1_	0.278	2	C11	0.309	0.086	1
			C12	0.232	0.065	7
			C13	0.214	0.060	8
			C14	0.245	0.068	6
D_2_	0.199	3	C21	0.270	0.054	11
			C22	0.213	0.042	14
			C23	0.239	0.047	12
			C24	0.278	0.055	10
D_3_	0.159	4	C31	0.445	0.071	5
			C32	0.259	0.041	15
			C33	0.296	0.047	13
D_4_	0.364	1	C41	0.227	0.083	2
			C42	0.227	0.083	3
			C43	0.203	0.074	4
			C44	0.099	0.036	16
			C45	0.154	0.056	9
			C46	0.090	0.033	17

## Conclusions and discussion

Integrating environmental and social sustainability into business processes is becoming crucial to remain competitive in the current business environment. However, contemporary literature focusing on food service industry performance measurement is solely based on economic indicators and falls short in integrating these considerations into the evaluation process. Aiming at filling this gap, this study proposed an integrated approach combining social, environmental, and economic aspects together in the performance evaluation system of a fast food restaurant company. A Balanced Scorecard based Grey-DANP approach is applied to reveal the influences among the evaluation criteria and rank them with respect to their importance weights. A fast food restaurant company is selected as a case study to demonstrate the applicability of the approach.

Besides being the earliest study in combining Balanced Scorecard and Grey-DANP approaches in integrating sustainability aspects into performance evaluation framework, this research identifies the environmental and social performance measures a food store could utilize. Previously published studies consider the sustainability measures independent from one another and hence, mostly avoid the inter-influences which are not compatible with real world applications. Moreover, this study highlights that avoiding these criteria interactions may result in misleading criteria weights. The proposed approach revealed that although financial measures had higher importance values, environmental, and social measures also had significant influence. For instance, as it can be observed from Table 4 and Figure 2A, Financial Perspective (D1) has stronger relationships with the remaining BSC dimensions while Customer Perspective (D3) has the highest influence on others. As far as the evaluation criteria under the BSC perspectives are concerned, similar findings can be observed from Figure 2 and Table 4. The final ranking of the evaluation criteria with respect to their importance level is provided in Table 5. In terms of managerial implications, the findings of the proposed approach can provide some insight that can guide the company management to improve the store performance based on the criteria that have significant influence on the performance (18). In the future, the results of this study can be utilized in a performance evaluation technique (e.g., TOPSIS) with numerical data collected from the stores. This data can then be used to rank the stores with respect to their individual performances. The criteria set can be expanded to include additional environmental and social measures such as reduction in water and energy consumption in each store. Furthermore, for comparison purposes, a conventional AHP based study could be conducted to obtain the criteria weights in the same case study.

## Author contributions

GD and EK devised the project, the main conceptual ideas and proof outline. GD worked out almost all of the technical details, and performed the numerical calculations for the case study. MT provided the data and information regarding the case study. EK and KR verified the analytical methods. All authors provided critical feedback and helped shape the research and analysis. All authors discussed the results and contributed to the final manuscript.

### Conflict of interest statement

The authors declare that the research was conducted in the absence of any commercial or financial relationships that could be construed as a potential conflict of interest.

## References

[1] USDA Food Accounts for 12.6 Percent of American Households' Expenditures (2017). Available online at: https://www.ers.usda.gov/data-products/chart-gallery/gallery/chart-detail/?chartId=58276

[2] USBEA GDP-by-Industry Data. U.S. Bureau of Economic Analysis (2017). Available online at: https://bea.gov/iTable/index_industry_gdpIndy.cfm (Accessed November 2, 2017).

[3] Alvarez A Fast Food Restaurants in the US. (2016). Available online at: www.ibisworld.com

[4] St. Jude Join Domino's in Supporting St. Jude. (2017). Available online at: https://www.stjude.org/get-involved/other-ways/partner-with-st-jude/corporate-partners/domino-s-pizza.html

[5] Ben&Jerry Fairtrade (2017). Available online at: https://www.benjerry.com/values/issues-we-care-about/fairtrade

[6] Starbucks Global Responsibility Report (2014). Available online at: https://news.starbucks.com/uploads/documents/FY14_GR_Report_British_English_Translation.pdf

[7] Petrini M Pozzebon M Integrating sustainability into business practices: learning from Brazilian firms. Braz Administr Rev. (2010) 7:362–78. 10.1590/S1807-76922010000400004

[8] Duman GM Tozanli O Kongar E Gupta SM A holistic approach for performance evaluation using quantitative and qualitative data: a food industry case study. Expert Syst Appl. (2017) 81:410–22. 10.1016/j.eswa.2017.03.070

[9] Gerbens-Leenes PW Moll HC Schoot Uiterkamp AJM Design and development of a measuring method for environmental sustainability in food production systems. Ecol Econ. (2003) 46:231–48. 10.1016/S0921-8009(03)00140-X

[10] Salvá M Jones S Marshall RJ Bishop CFH An audit tool for environmental measurement in the UK food sector. Int J Food Sci Technol. (2013) 48:1509–18. 10.1111/ijfs.12119

[11] Maloni MJ Brown ME Corporate social responsibility in the supply chain: an application in the food industry. J Business Ethics (2006) 68:35–52. 10.1007/s10551-006-9038-0

[12] Hartmann M Corporate social responsibility in the food sector. Eur Rev Agricult Econ. (2011) 38:297–324. 10.1093/erae/jbr031

[13] Kaplan RS Norton DP Putting the balanced scorecard to work. Perform Measur Manag Appraisal Sourcebook (1995) 66:17511.

[14] Epstein MJ Wisner P Good neighbors: implementing social and environmental strategies with the BSC. Balanced Scorecard Report. Reprint Number B0105C, Boston, MA: Harvard Business School Publishing (2001) 3:8–11.

[15] Figge F Hahn T Schaltegger S Wagner M The Sustainability Balanced Scorecard – linking sustainability management to business strategy. Business Strat Environ. (2002) 11:269–84. 10.1002/bse.339

[16] Hervani AA Helms MM Sarkis J Performance measurement for green supply chain management. Benchmarking (2005) 12:330–53. 10.1108/14635770510609015

[17] Kongar E Performance measurement for supply chain management and evaluation criteria determination for reverse supply chain management. Paper Presented at the SPIE International Conference on Environmentally Conscious Manufacturing. Philadelphia, PA (2004).

[18] Tsai WH Chou WC Hsu W The sustainability balanced scorecard as a framework for selecting socially responsible investment: an effective MCDM model. J Operation Res Soc. (2009) 60:1396–410. 10.1057/jors.2008.91

[19] Hsu CW Hu AH Chiou CY Chen TC Using the FDM and ANP to construct a sustainability balanced scorecard for the semiconductor industry. Expert Syst Appl. (2011) 38:12891–9. 10.1016/j.eswa.2011.04.082

[20] Bhattacharya A Mohapatra P Kumar V Dey PK Brady M Tiwari MK Green supply chain performance measurement using fuzzy ANP-based balanced scorecard: a collaborative decision-making approach. Prod Plan Control (2014) 25:698–714. 10.1080/09537287.2013.798088

[21] Rabbani A Zamani M Yazdani-Chamzini A Zavadskas EK Proposing a new integrated model based on sustainability balanced scorecard (SBSC) and MCDM approaches by using linguistic variables for the performance evaluation of oil producing companies. Expert Syst Appl. (2014) 41:7316–27. 10.1016/j.eswa.2014.05.023

[22] Gabus A Fontela E World Problems, an Invitation to Further Thought Within the Framework of DEMATEL. Geneva: Battelle Geneva Research Center (1972).

[23] Gabus A Fontela E Perceptions of the World Problematique: Communication Procedure, Communicating With Those Bearing Collective Responsibility. Geneva: Battelle Geneva Research Centre (1973).

[24] Saaty TL Decision Making With Dependence and Feedback: The Analytic Network Process Vol. 4922. Pittsburgh: RWS publications (1996).

[25] Büyüközkan G Güleryüz S An integrated DEMATEL-ANP approach for renewable energy resources selection in Turkey. Int J Prod Econ. (2016) 182:435–48. 10.1016/j.ijpe.2016.09.015

[26] Gölcük I Baykasoglu A An analysis of DEMATEL approaches for criteria interaction handling within ANP. Expert Syst Appl. (2016) 46:346–66. 10.1016/j.eswa.2015.10.041

[27] Chen FH Hsu TS Tzeng GH A balanced scorecard approach to establish a performance evaluation and relationship model for hot spring hotels based on a hybrid MCDM model combining DEMATEL and ANP. Int J Hosp Manag. (2011) 30:908–32. 10.1016/j.ijhm.2011.02.001

[28] Chiu WY Tzeng GH Li HL A new hybrid MCDM model combining DANP with VIKOR to improve e-store business. Knowled Based Syst. (2013) 37:48–61. 10.1016/j.knosys.2012.06.017

[29] Hsu CH Wang FK Tzeng GH The best vendor selection for conducting the recycled material based on a hybrid MCDM model combining DANP with VIKOR. Resour Conserv Recycl. (2012) 66:95–111. 10.1016/j.resconrec.2012.02.009

[30] Hung YH Huang TL Hsieh JC Tsuei HJ Cheng CC Tzeng GH Online reputation management for improving marketing by using a hybrid MCDM model. Knowled Based Syst. (2012) 35:87–93. 10.1016/j.knosys.2012.03.004

[31] Lee WS Huang AY Chang YY Cheng CM Analysis of decision making factors for equity investment by DEMATEL and Analytic Network Process. Expert Syst Appl. (2011) 38:8375–83. 10.1016/j.eswa.2011.01.027

[32] Liou JJ Building an effective system for carbon reduction management. J Clean Prod. (2015) 103:353–61. 10.1016/j.jclepro.2014.10.053

[33] Wu WW Choosing knowledge management strategies by using a combined ANP and DEMATEL approach. Expert Syst Appl. (2008) 35:828–35. 10.1016/j.eswa.2007.07.025

[34] Wu WW Lee YT Developing global managers' competencies using the fuzzy DEMATEL method. Expert Syst Appl. (2007) 32:499–507. 10.1016/j.eswa.2005.12.005

[35] Deng JL Control problems of grey systems. Syst Control Lett. (1982) 1:288–94. 10.1016/S0167-6911(82)80025-X

[36] Fu X Zhu Q Sarkis J Evaluating green supplier development programs at a telecommunications systems provider. Int J Prod Econ. (2012) 140:357–67. 10.1016/j.ijpe.2011.08.030

[37] Dou Y Zhu Q Sarkis J Evaluating green supplier development programs with a grey-analytical network process-based methodology. Eur J Operat Res. (2014) 233:420–31. 10.1016/j.ejor.2013.03.004

[38] Hsu CI Wen YH Application of grey theory and multiobjective programming towards airline network design. Eur J Operat Res. (2000) 127:44–68. 10.1016/S0377-2217(99)00320-3

[39] Tozanli O Duman G Kongar E Gupta S Environmentally concerned logistics operations in fuzzy environment: a literature survey. Logistics (2017) 1:4 10.3390/logistics1010004

[40] Golmohammadi D Mellat-Parast M Developing a grey-based decision-making model for supplier selection. Int J Prod Econ. (2012) 137:191–200. 10.1016/j.ijpe.2012.01.025

[41] Chithambaranathan P Subramanian N Gunasekaran A Palaniappan PK Service supply chain environmental performance evaluation using grey based hybrid MCDM approach. Int J Prod Econ. (2015) 166:163–76. 10.1016/j.ijpe.2015.01.002

[42] Çelikbilek Y Tüysüz F An integrated grey based multi-criteria decision making approach for the evaluation of renewable energy sources. Energy (2016) 115:1246–58. 10.1016/j.energy.2016.09.091

[43] Kaplan RS Norton DP Strategy Maps: Converting Intangible Assets Into Tangible Outcomes. Boston, MA: Harvard Business Press (2004).

[44] Opricovic S Tzeng GH Defuzzification within a multicriteria decision model. Int J Uncert Fuzziness Knowled Based Syst. (2003) 11:635–52. 10.1142/s0218488503002387

[45] Huang CN Liou JJ Chuang Y A method for exploring the interdependencies and importance of critical infrastructures. Knowled Based Syst. (2014) 55:66–74. 10.1016/j.knosys.2013.10.010

