# Supplementary material for: Integrating Environmental and Social Sustainability Into Performance Evaluation: A Balanced Scorecard-Based Grey-DANP Approach for the Food Industry
Source: Front Nutr. 2018 Jul 20;5:65. doi: 10.3389/fnut.2018.00065 (PMC6064944; doi:10.3389/fnut.2018.00065)
Supplement: Supplementary file 1 [file Data_Sheet_1.docx]

**Appendix A**

**Table A1.** The initial direct relation matrix

| Criteria | $C_{11}$ | $C_{12}$ | $C_{13}$ | $C_{14}$ | $C_{21}$ | $C_{22}$ | $C_{23}$ | $C_{24}$ | $C_{31}$ | $C_{32}$ | $C_{33}$ | $C_{41}$ | $C_{42}$ | $C_{43}$ | $C_{44}$ | $C_{45}$ | $C_{46}$ |
| --- | --- | --- | --- | --- | --- | --- | --- | --- | --- | --- | --- | --- | --- | --- | --- | --- | --- |
| $C_{11}$ | 0.00 | 0.69 | 0.95 | 0.95 | 0.00 | 0.00 | 0.00 | 0.00 | 0.00 | 0.00 | 0.00 | 0.95 | 0.95 | 0.95 | 0.69 | 0.35 | 0.00 |
| $C_{12}$ | 0.95 | 0.00 | 0.65 | 0.65 | 0.06 | 0.06 | 0.06 | 0.05 | 0.00 | 0.35 | 0.65 | 0.65 | 0.65 | 0.00 | 0.00 | 0.05 | 0.00 |
| $C_{13}$ | 0.95 | 0.38 | 0.00 | 0.00 | 0.06 | 0.06 | 0.06 | 0.05 | 0.00 | 0.05 | 0.05 | 0.95 | 0.95 | 0.65 | 0.06 | 0.65 | 0.00 |
| $C_{14}$ | 0.95 | 0.38 | 0.00 | 0.00 | 0.06 | 0.06 | 0.06 | 0.05 | 0.00 | 0.05 | 0.05 | 0.95 | 0.95 | 0.65 | 0.06 | 0.65 | 0.00 |
| $C_{21}$ | 0.00 | 0.38 | 0.00 | 0.00 | 0.00 | 0.38 | 0.69 | 0.65 | 0.65 | 0.35 | 0.35 | 0.00 | 0.00 | 0.00 | 0.38 | 0.35 | 0.35 |
| $C_{22}$ | 0.00 | 0.38 | 0.00 | 0.00 | 0.38 | 0.00 | 0.69 | 0.65 | 0.35 | 0.35 | 0.35 | 0.00 | 0.00 | 0.00 | 0.06 | 0.35 | 0.05 |
| $C_{23}$ | 0.95 | 0.38 | 0.00 | 0.00 | 0.69 | 0.69 | 0.00 | 0.65 | 0.65 | 0.35 | 0.35 | 0.00 | 0.00 | 0.00 | 0.06 | 0.05 | 0.05 |
| $C_{24}$ | 0.00 | 0.06 | 0.00 | 0.00 | 0.69 | 0.69 | 0.69 | 0.00 | 0.05 | 0.35 | 0.35 | 0.00 | 0.00 | 0.05 | 0.06 | 0.35 | 0.35 |
| $C_{31}$ | 0.00 | 0.00 | 0.65 | 0.65 | 0.69 | 0.69 | 0.69 | 0.65 | 0.00 | 0.05 | 0.05 | 0.95 | 0.95 | 0.65 | 0.38 | 0.00 | 0.05 |
| $C_{32}$ | 0.00 | 0.06 | 0.05 | 0.05 | 0.38 | 0.38 | 0.38 | 0.65 | 0.00 | 0.00 | 0.65 | 0.00 | 0.00 | 0.00 | 0.06 | 0.05 | 0.95 |
| $C_{33}$ | 0.00 | 0.06 | 0.05 | 0.05 | 0.69 | 0.38 | 0.69 | 0.65 | 0.05 | 0.95 | 0.00 | 0.00 | 0.00 | 0.00 | 0.06 | 0.95 | 0.95 |
| $C_{41}$ | 0.95 | 0.38 | 0.95 | 0.95 | 0.00 | 0.00 | 0.00 | 0.00 | 0.95 | 0.00 | 0.00 | 0.00 | 0.95 | 0.95 | 0.06 | 0.35 | 0.00 |
| $C_{42}$ | 0.95 | 0.38 | 0.95 | 0.95 | 0.00 | 0.00 | 0.00 | 0.00 | 0.95 | 0.00 | 0.00 | 0.95 | 0.00 | 0.95 | 0.06 | 0.35 | 0.00 |
| $C_{43}$ | 0.95 | 0.38 | 0.00 | 0.65 | 0.00 | 0.00 | 0.00 | 0.00 | 0.95 | 0.35 | 0.00 | 0.95 | 0.95 | 0.00 | 0.06 | 0.05 | 0.05 |
| $C_{44}$ | 0.05 | 0.69 | 0.00 | 0.00 | 0.69 | 0.38 | 0.06 | 0.05 | 0.05 | 0.05 | 0.05 | 0.00 | 0.00 | 0.35 | 0.00 | 0.00 | 0.05 |
| $C_{45}$ | 0.05 | 0.38 | 0.05 | 0.05 | 0.69 | 0.06 | 0.06 | 0.65 | 0.05 | 0.05 | 0.95 | 0.00 | 0.00 | 0.35 | 0.69 | 0.00 | 0.00 |
| $C_{46}$ | 0.00 | 0.06 | 0.00 | 0.00 | 0.06 | 0.06 | 0.38 | 0.95 | 0.95 | 0.95 | 0.95 | 0.00 | 0.00 | 0.00 | 0.00 | 0.00 | 0.00 |

**Table A2.** The total relation matrix

| Criteria | $C_{11}$ | $C_{12}$ | $C_{13}$ | $C_{14}$ | $C_{21}$ | $C_{22}$ | $C_{23}$ | $C_{24}$ | $C_{31}$ | $C_{32}$ | $C_{33}$ | $C_{41}$ | $C_{42}$ | $C_{43}$ | $C_{44}$ | $C_{45}$ | $C_{46}$ |
| --- | --- | --- | --- | --- | --- | --- | --- | --- | --- | --- | --- | --- | --- | --- | --- | --- | --- |
| $C_{11}$ | 0.31 | 0.28 | 0.34 | 0.38 | 0.10 | 0.07 | 0.07 | 0.09 | 0.20 | 0.08 | 0.09 | 0.44 | 0.44 | 0.40 | 0.18 | 0.20 | 0.04 |
| $C_{12}$ | 0.34 | 0.13 | 0.25 | 0.26 | 0.09 | 0.07 | 0.08 | 0.09 | 0.14 | 0.12 | 0.16 | 0.30 | 0.30 | 0.20 | 0.07 | 0.14 | 0.05 |
| $C_{13}$ | 0.36 | 0.20 | 0.18 | 0.21 | 0.09 | 0.07 | 0.07 | 0.09 | 0.16 | 0.07 | 0.08 | 0.37 | 0.37 | 0.31 | 0.09 | 0.21 | 0.03 |
| $C_{14}$ | 0.36 | 0.20 | 0.18 | 0.21 | 0.09 | 0.07 | 0.07 | 0.09 | 0.16 | 0.07 | 0.08 | 0.37 | 0.37 | 0.31 | 0.09 | 0.21 | 0.03 |
| $C_{21}$ | 0.10 | 0.13 | 0.07 | 0.08 | 0.11 | 0.14 | 0.19 | 0.20 | 0.18 | 0.13 | 0.14 | 0.09 | 0.09 | 0.08 | 0.10 | 0.12 | 0.10 |
| $C_{22}$ | 0.08 | 0.11 | 0.05 | 0.06 | 0.14 | 0.07 | 0.17 | 0.18 | 0.12 | 0.11 | 0.12 | 0.07 | 0.07 | 0.06 | 0.05 | 0.11 | 0.06 |
| $C_{23}$ | 0.25 | 0.15 | 0.10 | 0.12 | 0.20 | 0.18 | 0.10 | 0.20 | 0.19 | 0.13 | 0.13 | 0.14 | 0.14 | 0.12 | 0.08 | 0.10 | 0.07 |
| $C_{24}$ | 0.07 | 0.07 | 0.04 | 0.04 | 0.18 | 0.16 | 0.17 | 0.10 | 0.08 | 0.12 | 0.13 | 0.05 | 0.05 | 0.05 | 0.05 | 0.11 | 0.10 |
| $C_{31}$ | 0.28 | 0.17 | 0.27 | 0.30 | 0.22 | 0.20 | 0.21 | 0.22 | 0.21 | 0.10 | 0.11 | 0.39 | 0.39 | 0.32 | 0.14 | 0.16 | 0.06 |
| $C_{32}$ | 0.06 | 0.06 | 0.04 | 0.05 | 0.13 | 0.12 | 0.14 | 0.19 | 0.08 | 0.08 | 0.17 | 0.05 | 0.05 | 0.04 | 0.04 | 0.07 | 0.19 |
| $C_{33}$ | 0.09 | 0.09 | 0.06 | 0.07 | 0.21 | 0.15 | 0.21 | 0.24 | 0.11 | 0.23 | 0.13 | 0.07 | 0.07 | 0.07 | 0.07 | 0.21 | 0.21 |
| $C_{41}$ | 0.45 | 0.24 | 0.36 | 0.40 | 0.11 | 0.08 | 0.09 | 0.10 | 0.33 | 0.08 | 0.09 | 0.35 | 0.46 | 0.42 | 0.12 | 0.21 | 0.04 |
| $C_{42}$ | 0.45 | 0.24 | 0.36 | 0.40 | 0.11 | 0.08 | 0.09 | 0.10 | 0.33 | 0.08 | 0.09 | 0.46 | 0.35 | 0.42 | 0.12 | 0.21 | 0.04 |
| $C_{43}$ | 0.39 | 0.21 | 0.21 | 0.33 | 0.09 | 0.08 | 0.08 | 0.09 | 0.31 | 0.11 | 0.08 | 0.41 | 0.41 | 0.25 | 0.10 | 0.14 | 0.05 |
| $C_{44}$ | 0.08 | 0.15 | 0.05 | 0.06 | 0.14 | 0.09 | 0.06 | 0.06 | 0.07 | 0.05 | 0.05 | 0.07 | 0.07 | 0.10 | 0.03 | 0.04 | 0.03 |
| $C_{45}$ | 0.09 | 0.12 | 0.06 | 0.07 | 0.18 | 0.08 | 0.09 | 0.18 | 0.08 | 0.08 | 0.20 | 0.08 | 0.08 | 0.11 | 0.14 | 0.08 | 0.06 |
| $C_{46}$ | 0.08 | 0.07 | 0.06 | 0.07 | 0.12 | 0.10 | 0.16 | 0.25 | 0.21 | 0.22 | 0.22 | 0.09 | 0.09 | 0.07 | 0.05 | 0.08 | 0.08 |

**Table A3.** The unweighted super-matrix

| Criteria | $C_{11}$ | $C_{12}$ | $C_{13}$ | $C_{14}$ | $C_{21}$ | $C_{22}$ | $C_{23}$ | $C_{24}$ | $C_{31}$ | $C_{32}$ | $C_{33}$ | $C_{41}$ | $C_{42}$ | $C_{43}$ | $C_{44}$ | $C_{45}$ | $C_{46}$ |
| --- | --- | --- | --- | --- | --- | --- | --- | --- | --- | --- | --- | --- | --- | --- | --- | --- | --- |
| $C_{11}$ | 0.241 | 0.346 | 0.381 | 0.381 | 0.265 | 0.265 | 0.403 | 0.302 | 0.271 | 0.279 | 0.286 | 0.310 | 0.310 | 0.343 | 0.242 | 0.262 | 0.286 |
| $C_{12}$ | 0.212 | 0.136 | 0.208 | 0.208 | 0.346 | 0.369 | 0.244 | 0.316 | 0.171 | 0.291 | 0.296 | 0.167 | 0.167 | 0.184 | 0.431 | 0.353 | 0.251 |
| $C_{13}$ | 0.260 | 0.250 | 0.191 | 0.191 | 0.185 | 0.174 | 0.168 | 0.180 | 0.265 | 0.206 | 0.199 | 0.248 | 0.248 | 0.186 | 0.150 | 0.177 | 0.220 |
| $C_{14}$ | 0.288 | 0.269 | 0.220 | 0.220 | 0.204 | 0.192 | 0.185 | 0.202 | 0.294 | 0.224 | 0.219 | 0.275 | 0.275 | 0.287 | 0.177 | 0.207 | 0.244 |
| $C_{21}$ | 0.301 | 0.275 | 0.290 | 0.290 | 0.169 | 0.247 | 0.290 | 0.288 | 0.262 | 0.230 | 0.266 | 0.285 | 0.285 | 0.275 | 0.404 | 0.347 | 0.192 |
| $C_{22}$ | 0.213 | 0.210 | 0.210 | 0.210 | 0.217 | 0.127 | 0.264 | 0.261 | 0.234 | 0.205 | 0.182 | 0.215 | 0.215 | 0.219 | 0.259 | 0.148 | 0.164 |
| $C_{23}$ | 0.218 | 0.238 | 0.225 | 0.225 | 0.297 | 0.305 | 0.152 | 0.285 | 0.246 | 0.236 | 0.257 | 0.228 | 0.228 | 0.233 | 0.163 | 0.172 | 0.251 |
| $C_{24}$ | 0.268 | 0.277 | 0.275 | 0.275 | 0.316 | 0.320 | 0.295 | 0.166 | 0.258 | 0.329 | 0.294 | 0.272 | 0.272 | 0.273 | 0.174 | 0.333 | 0.392 |
| $C_{31}$ | 0.547 | 0.330 | 0.518 | 0.518 | 0.403 | 0.336 | 0.425 | 0.256 | 0.493 | 0.234 | 0.241 | 0.660 | 0.660 | 0.613 | 0.395 | 0.231 | 0.330 |
| $C_{32}$ | 0.208 | 0.281 | 0.217 | 0.217 | 0.287 | 0.318 | 0.280 | 0.360 | 0.246 | 0.252 | 0.490 | 0.158 | 0.158 | 0.228 | 0.290 | 0.225 | 0.335 |
| $C_{33}$ | 0.244 | 0.389 | 0.265 | 0.265 | 0.310 | 0.345 | 0.296 | 0.384 | 0.261 | 0.513 | 0.270 | 0.182 | 0.182 | 0.158 | 0.315 | 0.544 | 0.335 |
| $C_{41}$ | 0.257 | 0.284 | 0.266 | 0.266 | 0.155 | 0.167 | 0.215 | 0.126 | 0.266 | 0.109 | 0.102 | 0.216 | 0.290 | 0.300 | 0.204 | 0.145 | 0.190 |
| $C_{42}$ | 0.257 | 0.284 | 0.266 | 0.266 | 0.155 | 0.167 | 0.215 | 0.126 | 0.266 | 0.109 | 0.102 | 0.290 | 0.216 | 0.300 | 0.204 | 0.145 | 0.190 |
| $C_{43}$ | 0.234 | 0.185 | 0.222 | 0.222 | 0.135 | 0.144 | 0.186 | 0.130 | 0.220 | 0.098 | 0.098 | 0.262 | 0.262 | 0.185 | 0.287 | 0.211 | 0.164 |
| $C_{44}$ | 0.109 | 0.070 | 0.068 | 0.068 | 0.170 | 0.119 | 0.119 | 0.116 | 0.096 | 0.093 | 0.095 | 0.073 | 0.073 | 0.074 | 0.083 | 0.251 | 0.100 |
| $C_{45}$ | 0.120 | 0.130 | 0.152 | 0.152 | 0.205 | 0.264 | 0.161 | 0.260 | 0.107 | 0.160 | 0.301 | 0.133 | 0.133 | 0.106 | 0.129 | 0.139 | 0.179 |
| $C_{46}$ | 0.022 | 0.047 | 0.025 | 0.025 | 0.179 | 0.139 | 0.104 | 0.242 | 0.045 | 0.431 | 0.302 | 0.025 | 0.025 | 0.035 | 0.095 | 0.108 | 0.178 |

**Table A4.** The weighted super-matrix

| Criteria | $C_{11}$ | $C_{12}$ | $C_{13}$ | $C_{14}$ | $C_{21}$ | $C_{22}$ | $C_{23}$ | $C_{24}$ | $C_{31}$ | $C_{32}$ | $C_{33}$ | $C_{41}$ | $C_{42}$ | $C_{43}$ | $C_{44}$ | $C_{45}$ | $C_{46}$ |
| --- | --- | --- | --- | --- | --- | --- | --- | --- | --- | --- | --- | --- | --- | --- | --- | --- | --- |
| $C_{11}$ | 0.082 | 0.117 | 0.129 | 0.129 | 0.053 | 0.053 | 0.080 | 0.060 | 0.055 | 0.056 | 0.058 | 0.096 | 0.096 | 0.106 | 0.075 | 0.081 | 0.088 |
| $C_{12}$ | 0.072 | 0.046 | 0.070 | 0.070 | 0.069 | 0.073 | 0.049 | 0.063 | 0.035 | 0.059 | 0.060 | 0.052 | 0.052 | 0.057 | 0.133 | 0.109 | 0.077 |
| $C_{13}$ | 0.088 | 0.085 | 0.065 | 0.065 | 0.037 | 0.035 | 0.033 | 0.036 | 0.054 | 0.042 | 0.040 | 0.077 | 0.077 | 0.057 | 0.046 | 0.055 | 0.068 |
| $C_{14}$ | 0.097 | 0.091 | 0.075 | 0.075 | 0.041 | 0.038 | 0.037 | 0.040 | 0.059 | 0.045 | 0.044 | 0.085 | 0.085 | 0.089 | 0.055 | 0.064 | 0.075 |
| $C_{21}$ | 0.031 | 0.028 | 0.030 | 0.030 | 0.055 | 0.080 | 0.094 | 0.094 | 0.077 | 0.068 | 0.078 | 0.046 | 0.046 | 0.044 | 0.065 | 0.056 | 0.031 |
| $C_{22}$ | 0.022 | 0.022 | 0.022 | 0.022 | 0.071 | 0.041 | 0.086 | 0.085 | 0.069 | 0.060 | 0.054 | 0.035 | 0.035 | 0.035 | 0.042 | 0.024 | 0.026 |
| $C_{23}$ | 0.022 | 0.024 | 0.023 | 0.023 | 0.096 | 0.099 | 0.049 | 0.092 | 0.073 | 0.069 | 0.076 | 0.037 | 0.037 | 0.038 | 0.026 | 0.028 | 0.041 |
| $C_{24}$ | 0.028 | 0.028 | 0.028 | 0.028 | 0.103 | 0.104 | 0.096 | 0.054 | 0.076 | 0.097 | 0.087 | 0.044 | 0.044 | 0.044 | 0.028 | 0.054 | 0.063 |
| $C_{31}$ | 0.062 | 0.038 | 0.059 | 0.059 | 0.084 | 0.070 | 0.088 | 0.053 | 0.080 | 0.038 | 0.039 | 0.109 | 0.109 | 0.102 | 0.065 | 0.038 | 0.055 |
| $C_{32}$ | 0.024 | 0.032 | 0.025 | 0.025 | 0.059 | 0.066 | 0.058 | 0.075 | 0.040 | 0.041 | 0.079 | 0.026 | 0.026 | 0.038 | 0.048 | 0.037 | 0.055 |
| $C_{33}$ | 0.028 | 0.044 | 0.030 | 0.030 | 0.064 | 0.072 | 0.061 | 0.080 | 0.042 | 0.083 | 0.044 | 0.030 | 0.030 | 0.026 | 0.052 | 0.090 | 0.056 |
| $C_{41}$ | 0.114 | 0.126 | 0.118 | 0.118 | 0.042 | 0.045 | 0.058 | 0.034 | 0.091 | 0.037 | 0.035 | 0.079 | 0.106 | 0.109 | 0.074 | 0.053 | 0.069 |
| $C_{42}$ | 0.114 | 0.126 | 0.118 | 0.118 | 0.042 | 0.045 | 0.058 | 0.034 | 0.091 | 0.037 | 0.035 | 0.106 | 0.079 | 0.109 | 0.074 | 0.053 | 0.069 |
| $C_{43}$ | 0.104 | 0.082 | 0.099 | 0.099 | 0.036 | 0.039 | 0.050 | 0.035 | 0.075 | 0.033 | 0.033 | 0.095 | 0.095 | 0.068 | 0.104 | 0.077 | 0.060 |
| $C_{44}$ | 0.048 | 0.031 | 0.030 | 0.030 | 0.046 | 0.032 | 0.032 | 0.031 | 0.033 | 0.032 | 0.033 | 0.027 | 0.027 | 0.027 | 0.030 | 0.091 | 0.036 |
| $C_{45}$ | 0.053 | 0.058 | 0.068 | 0.068 | 0.055 | 0.071 | 0.043 | 0.070 | 0.037 | 0.055 | 0.103 | 0.049 | 0.049 | 0.039 | 0.047 | 0.051 | 0.065 |
| $C_{46}$ | 0.010 | 0.021 | 0.011 | 0.011 | 0.048 | 0.037 | 0.028 | 0.065 | 0.015 | 0.147 | 0.103 | 0.009 | 0.009 | 0.013 | 0.034 | 0.040 | 0.065 |

**Table A5.** The limit super-matrix

| Criteria | $C_{11}$ | $C_{12}$ | $C_{13}$ | $C_{14}$ | $C_{21}$ | $C_{22}$ | $C_{23}$ | $C_{24}$ | $C_{31}$ | $C_{32}$ | $C_{33}$ | $C_{41}$ | $C_{42}$ | $C_{43}$ | $C_{44}$ | $C_{45}$ | $C_{46}$ |
| --- | --- | --- | --- | --- | --- | --- | --- | --- | --- | --- | --- | --- | --- | --- | --- | --- | --- |
| $C_{11}$ | 0.086 | 0.086 | 0.086 | 0.086 | 0.086 | 0.086 | 0.086 | 0.086 | 0.086 | 0.086 | 0.086 | 0.086 | 0.086 | 0.086 | 0.086 | 0.086 | 0.086 |
| $C_{12}$ | 0.065 | 0.065 | 0.065 | 0.065 | 0.065 | 0.065 | 0.065 | 0.065 | 0.065 | 0.065 | 0.065 | 0.065 | 0.065 | 0.065 | 0.065 | 0.065 | 0.065 |
| $C_{13}$ | 0.060 | 0.060 | 0.060 | 0.060 | 0.060 | 0.060 | 0.060 | 0.060 | 0.060 | 0.060 | 0.060 | 0.060 | 0.060 | 0.060 | 0.060 | 0.060 | 0.060 |
| $C_{14}$ | 0.068 | 0.068 | 0.068 | 0.068 | 0.068 | 0.068 | 0.068 | 0.068 | 0.068 | 0.068 | 0.068 | 0.068 | 0.068 | 0.068 | 0.068 | 0.068 | 0.068 |
| $C_{21}$ | 0.054 | 0.054 | 0.054 | 0.054 | 0.054 | 0.054 | 0.054 | 0.054 | 0.054 | 0.054 | 0.054 | 0.054 | 0.054 | 0.054 | 0.054 | 0.054 | 0.054 |
| $C_{22}$ | 0.042 | 0.042 | 0.042 | 0.042 | 0.042 | 0.042 | 0.042 | 0.042 | 0.042 | 0.042 | 0.042 | 0.042 | 0.042 | 0.042 | 0.042 | 0.042 | 0.042 |
| $C_{23}$ | 0.047 | 0.047 | 0.047 | 0.047 | 0.047 | 0.047 | 0.047 | 0.047 | 0.047 | 0.047 | 0.047 | 0.047 | 0.047 | 0.047 | 0.047 | 0.047 | 0.047 |
| $C_{24}$ | 0.055 | 0.055 | 0.055 | 0.055 | 0.055 | 0.055 | 0.055 | 0.055 | 0.055 | 0.055 | 0.055 | 0.055 | 0.055 | 0.055 | 0.055 | 0.055 | 0.055 |
| $C_{31}$ | 0.071 | 0.071 | 0.071 | 0.071 | 0.071 | 0.071 | 0.071 | 0.071 | 0.071 | 0.071 | 0.071 | 0.071 | 0.071 | 0.071 | 0.071 | 0.071 | 0.071 |
| $C_{32}$ | 0.041 | 0.041 | 0.041 | 0.041 | 0.041 | 0.041 | 0.041 | 0.041 | 0.041 | 0.041 | 0.041 | 0.041 | 0.041 | 0.041 | 0.041 | 0.041 | 0.041 |
| $C_{33}$ | 0.047 | 0.047 | 0.047 | 0.047 | 0.047 | 0.047 | 0.047 | 0.047 | 0.047 | 0.047 | 0.047 | 0.047 | 0.047 | 0.047 | 0.047 | 0.047 | 0.047 |
| $C_{41}$ | 0.083 | 0.083 | 0.083 | 0.083 | 0.083 | 0.083 | 0.083 | 0.083 | 0.083 | 0.083 | 0.083 | 0.083 | 0.083 | 0.083 | 0.083 | 0.083 | 0.083 |
| $C_{42}$ | 0.083 | 0.083 | 0.083 | 0.083 | 0.083 | 0.083 | 0.083 | 0.083 | 0.083 | 0.083 | 0.083 | 0.083 | 0.083 | 0.083 | 0.083 | 0.083 | 0.083 |
| $C_{43}$ | 0.074 | 0.074 | 0.074 | 0.074 | 0.074 | 0.074 | 0.074 | 0.074 | 0.074 | 0.074 | 0.074 | 0.074 | 0.074 | 0.074 | 0.074 | 0.074 | 0.074 |
| $C_{44}$ | 0.036 | 0.036 | 0.036 | 0.036 | 0.036 | 0.036 | 0.036 | 0.036 | 0.036 | 0.036 | 0.036 | 0.036 | 0.036 | 0.036 | 0.036 | 0.036 | 0.036 |
| $C_{45}$ | 0.056 | 0.056 | 0.056 | 0.056 | 0.056 | 0.056 | 0.056 | 0.056 | 0.056 | 0.056 | 0.056 | 0.056 | 0.056 | 0.056 | 0.056 | 0.056 | 0.056 |
| $C_{46}$ | 0.033 | 0.033 | 0.033 | 0.033 | 0.033 | 0.033 | 0.033 | 0.033 | 0.033 | 0.033 | 0.033 | 0.033 | 0.033 | 0.033 | 0.033 | 0.033 | 0.033 |
